# Comprehensive Evaluation of Elexacaftor/Tezacaftor/Ivacaftor in Paediatric Cystic Fibrosis: Nutritional, Pulmonary, and Quality-of-Life Outcomes †

**DOI:** 10.3390/jcm14227969

**Published:** 2025-11-10

**Authors:** Katarzyna Walicka-Serzysko, Magdalena Postek, Monika Mielus, Urszula Borawska-Kowalczyk, Justyna Milczewska, Katarzyna Zybert, Łukasz Wozniacki, Anna Wołkowicz, Dorota Sands

**Affiliations:** 1 Cystic Fibrosis Department, Institute of Mother and Child, 01-211 Warsaw, Poland; magdalena.postek@imid.med.pl (M.P.); monika.mielus@imid.med.pl (M.M.); urszula.borawska@imid.med.pl (U.B.-K.); justyna.milczewska@imid.med.pl (J.M.); katarzyna.zybert@imid.med.pl (K.Z.); lukasz.wozniacki@imid.med.pl (Ł.W.); anna.wolkowicz@imid.med.pl (A.W.); dorota.sands@imid.med.pl (D.S.); 2 Cystic Fibrosis Centre, Paediatric Hospital, 05-092 Dziekanów Leśny, Poland

**Keywords:** cystic fibrosis, CFTR modulator, spirometry, lung clearance index, health-related quality of life, nutritional status, body composition, fat mass, fat free mass

## Abstract

**Background/Objectives:** Cystic fibrosis transmembrane conductance regulator modulator (mCFTR) therapy has been proven efficacious in controlled clinical trials for individuals with cystic fibrosis. This post-approval retrospective study aimed to determine the comprehensive effects of elexacaftor/tezacaftor/ivacaftor (ETI) on nutritional status, the respiratory system and quality of life over 12 months of clinical use in paediatric patients treatment-naïve to mCFTR. **Methods**: A retrospective analysis of records of CF adolescents on ETI therapy was conducted. The selected parameters of anthropometric measurements, body composition assessed by BIA, spirometry, and multiple breath nitrogen washout (MBNW) to measure lung clearance index (LCI), were evaluated before therapy and at 3 and 12 months after treatment initiation. Additionally, children completed the Cystic Fibrosis Questionnaire-Revised (CFQ-R). **Results:** Over 18 months, data from 58 patients (mean age 14.34 ± 1.70, 50% female; 43% homozygous F508del) on ETI were collected. Body weight increased significantly over 12 months, with a mean gain of 3.33 kg at 3 months (*p* < 0.001) and 7.10 kg at 12 months (*p* < 0.001), alongside improvements in BMI z-score, fat-free mass, and fat mass. Significant changes (*p* < 0.001) were also observed after 3 and 12 months in ppFEV_1_ (8.91 ± 8.23; 9.67 ± 8.77) and ppFVC (4.46 ± 5.24; 4.61 ± 5.70), with a decrease in LCI (−1.62 ± 2.15; −1.68 ± 1.89). The CFQ-R Respiratory score increased by 11.75 points and correlated with most of the pulmonary and nutritional parameters. **Conclusions**: In real-world settings, clinical improvement during ETI therapy reflects a comprehensive impact on nutritional status, body composition, pulmonary function, and quality of life for adolescents with CF.

## 1. Background

Cystic fibrosis transmembrane conductance regulator modulator (mCFTR) therapy is the first variant-specific therapy (VST) tailored to the genetic defect that has brought transformative changes in cystic fibrosis (CF) care in recent years [1]. These small molecules restore the function of the CFTR channel, leading to improved nutritional status, lung function, and quality of life, as well as reduced pulmonary exacerbations (PEx), as demonstrated in clinical trials [2,3,4,5,6].

Nowadays, as more patients with cystic fibrosis (pwCF) receive VST since its introduction, it is crucial to evaluate the effectiveness and safety of this therapy in clinical practice. Research conducted in real-world settings shows that the benefits of treatment persist over time and that the most significant impact may be achieved through early implementation [7,8,9,10,11,12,13,14].

Improving nutritional status, supported by treatment addressing the underlying defect, proves the importance of monitoring anthropometric parameters and body composition in preventing overweight and obesity [1,15].

As a result of the improvement in the course of lung disease, the respiratory system’s response to VST assessed by spirometry becomes less sensitive in detecting early changes, especially in children and adults with normal spirometry. As clinical relevance and validity improve, more sensitive tests, such as multiple breath washout (MBW) to measure lung clearance index (LCI), are increasingly being used [1,16].

Additionally, assessment of patients’ health-related quality of life (HRQoL) constitutes a complementary and important factor in monitoring the effectiveness of treatment with mCFTR. It provides insight into whether the improvements observed through objective measures, such as laboratory tests and pulmonary function assessments, are also experienced subjectively by the patient. By capturing the patient’s perspective, HRQoL assessments offer valuable complementary information regarding the overall impact of therapy on daily functioning and well-being. Consequently, these measures are recognised as important endpoints in clinical trials, contributing to a more comprehensive evaluation of treatment efficacy [17]. Moreover, monitoring patients’ emotional well-being and HRQoL during mCFTR therapy is essential to assess their overall functioning and to provide timely and appropriate interventions in case of concerning symptoms [1,18].

According to European Cystic Fibrosis Society (ECFS) standards, due to irreversible changes occurring even in the first years of life, there is a focus on the importance of starting treatment for the underlying defect as early as possible [1].

In Europe, a highly effective modulator therapy (HEMT), the triple combination elexacaftor/tezacaftor/ivacaftor (ETI) was approved in August 2020 for treating pwCF aged ≥12 years who are F508del homozygous (F/F) or heterozygous with a minimal function variant (F/MF). Subsequently, approval was gradually extended to include all heterozygous individuals aged ≥12 years, then those aged ≥6 years, and now to those aged ≥2 years with at least one F508del variant. In Poland, the National Health Fund has been reimbursing ETI for pwCF aged ≥12 years with F/F and F/MF variants since 2022. Approval was extended to patients aged ≥2 years with at least one F508del variant only in 2025.

This single-centre study aimed to evaluate the effectiveness of ETI over a 12-month therapy period in adolescents who had not previously received VST. The impact on clinical status was assessed by analysing nutritional status (including anthropometric parameters and body composition), pulmonary function and health-related quality of life. A retrospective analysis was performed on selected pulmonary function parameters, including LCI, to detect early changes in lung disease, and their relationship with nutritional status and quality of life. This study was designed to address an important evidence gap by providing real-world paediatric data from a modulator-naïve cohort, uniquely characterised by the integrated analysis of nutrition, lung function, and quality of life within a defined national reimbursement context.

This study was presented as a poster at the European Cystic Fibrosis Conference in Glasgow, 5–8 June 2024 [19].

## 2. Methods

### 2.1. Study Design

This retrospective observational study was conducted at the Cystic Fibrosis Centre in Warsaw, which provides care for 300 children from infancy to 18 years of age. It was carried out following the principles outlined in the Declaration of Helsinki and good clinical practice. The local ethics committee at the Institute of Mother and Child accepted the trial protocol (No. 43/2024). All patients and their legal guardians gave written informed consent prior to participation in the study.

Between July 2022 and December 2023, we enrolled patients aged 12 to 17 with a confirmed diagnosis of CF based on current diagnostic criteria [18,20,21,22] and the rules of the Polish National Health Fund’s mCFTR programme. Reimbursement for ETI therapy was available for patients aged 12 and over with a homozygous F508del (F/F) or heterozygous F508del genotype and minimal function (F/MF). The inclusion criteria for our study also included stable clinical conditions, absence of haemoptysis, and other conditions preventing the ability to perform pulmonary function tests (PFTs). Patients who were unable to undergo PFTs were excluded from the study. The inclusion and exclusion criteria are presented in Table 1. Health-related quality of life was assessed using the Cystic Fibrosis Questionnaire-Revised (CFQ-R) specific questionnaire.

After obtaining written consent, a retrospective analysis of clinical assessments was performed during follow-up visits at the start of treatment and at 3 and 12 months. These evaluations included nutritional status, PFTs, quality of life, and emotional well-being. All examinations were conducted in accordance with cross-infection prevention standards in cystic fibrosis. Patients completed the CFQ-R questionnaire prior to other assessments to ensure that undergoing medical procedures did not influence their evaluation of quality of life.

### 2.2. Nutritional Status Measurements

Weight and height data were extracted from clinical records, and body mass index (BMI, kg/m^2^) was calculated. BMI was classified using z-scores for each age group and sex, based on reference values from the Centres for Disease Control and Prevention (CDC) to standardise data and enhance comparability. These values are included in the ECFS Patient Registry Data [23]. BMI z-scores were categorised according to the latest European nutrition guidelines [15]. Body composition analysis was performed using segmental multifrequency bioelectrical impedance analysis (BIA) with the Tanita MC-750 device. The following parameters were included in the analysis: fat-free mass (kg), fat-free mass index, fat mass (kg), and fat mass index. Compared to the gold standard for body composition assessment, dual-energy X-ray absorptiometry (DXA) using the four-component model, this BIA technology demonstrates close agreement across BMI levels in children [22]. BIA is also recommended by ESPEN-ESPGHAN-ECFS as a reliable tool for body composition assessment [15]. BIA measurements were performed under standardised conditions to minimise the influence of confounding factors such as food intake and hydration status. All measurements were conducted in the morning, when patients were in a fasting state and in normal hydration. This high level of standardisation was possible because BIA was integrated into the routine follow-up visit and performed concurrently with mandatory blood sampling for liver function monitoring within the national reimbursement programme. According to this standardised procedure, patients are required to fast for 8–10 h and to drink water according to recommendations, ensuring a consistent metabolic and hydration state, and thereby significantly increasing the reliability of BIA measurements.

### 2.3. Pulmonary Function Measurements

For the assessment of CF lung disease, alongside spirometry, the multiple breath nitrogen washout (MBNW) technique with assessment of LCI was utilised, providing the ability to detect changes at an early stage.

PFTs were performed according to the European Respiratory Society (ERS) guidelines. All equipment was calibrated every day before tests. During the calibration of the spirometer with a 3 L syringe, the error in measuring the linearity and repeatability did not exceed ±2.5%. Ambient temperature, barometric pressure, and air humidity were recorded every day before measurements.

The following morning, airway clearance therapy, patients underwent MBNW and spirometry. To prevent any impact on airway calibre from forced expiratory manoeuvres, MBNW was conducted before spirometry.

MBNW was performed using the Exhalyzer D device (EcoMedics AG, Duernten, Switzerland, software version 3.3.1). The test was seen as successful if at least two technically acceptable attempts followed the guidelines of the American Thoracic Society/European Respiratory Society (ATS/ERS) consensus statement [24]. Trials were deemed to be of high quality based not solely on software evaluation but also on careful observation of the subject’s behaviour during testing. LCI was expressed as the mean of a minimum of two technically acceptable results achieved during one session. The ULN of LCI ≥ 6.99 was determined based on normative data for MBNW with correction for sensor error [24,25].

Spirometry was conducted on all children according to the ATS/ERS criteria [26,27] using the Jaeger Vyntus IOS (CareFusion, Hochberg, Germany). Patients performed spirometry in a sitting position. They were required to accomplish at least three reproducible trials. Test results were considered reproducible if the difference between the two largest forced vital capacity (FVC) values was ≤0.100 L, there was no cough in the first second of expiration, no glottic closure after 1 s of expiration, no evidence of obstruction in the mouthpiece or spirometer, and no evidence of a leak reaching the expiratory plateau within 15 s [25,28,29].

Pulmonary function results were recorded and analysed according to the Global Lung Function Initiative (GLI) [28].

### 2.4. Health-Related Quality of Life Measurements

Health-related quality of life was assessed using the Polish version of the Cystic Fibrosis Questionnaire-Revised (CFQ-R), routinely administered during patients’ visits to the centre [30]. The CFQ-R is a widely used, validated, disease-specific instrument for QoL measurement in cystic fibrosis [31]. Two versions of the CFQ-R were used: *CFQ-R Child* for children aged 12–13 years and CFQ-R Teen/Adult for patients aged ≥14 years.

The questionnaire provides a summary score in each domain; scores range from 0 to 100, where higher scores indicate better QoL. Response choices include ratings of frequency and difficulty on a 4-point Likert scale. Three domains were chosen to be used in the study: Physical functioning, Eating problems, and Respiratory symptoms. The Physical functioning scale reflects the patient’s subjective evaluation of their physical condition, the Eating problems domain assesses the patient’s attitude toward meals and eating behaviours, and the Respiratory symptoms scale describes their pulmonary symptoms. The minimal clinically important difference (MCID) for the CFQ-R Respiratory scale is 4.0 points, which is the smallest difference in scores that patients perceive as clinically beneficial [32].

The above-mentioned pulmonary function indices, nutritional status parameters, as well as quality of life and emotional state, will undergo retrospective analysis, assessed both before the initiation of mCFTR therapy and at 3 and 12 months of its administration.

### 2.5. Statistical Analysis

Statistical analyses of PFT results and nutritional parameters were performed using Statistica software, version 13.3. Descriptive statistics were calculated, and the distribution of variables was assessed to determine the use of parametric (Student’s test) or non-parametric tests (Welch’s test, Wilcoxon’s test and Mann–Whitney’s test). The relationships between pulmonary function, nutritional indices, and selected parameters derived from QoL questionnaires were evaluated using Spearman’s rank correlation coefficient (Rho). Statistical significance was set at *p* < 0.05. Missing data were handled by pairwise complete cases: for each paired comparison (baseline vs. follow-up), we analysed participants with non-missing values at both time points for that endpoint.

The analysis of psychological questionnaires concerning quality of life was conducted with IBM SPSS Statistics, version 22. Descriptive statistics were calculated, and group comparisons were carried out with appropriate tests selected according to the distribution of the data. 

## 3. Results

### 3.1. Subjects’ Characteristics

**Figure 1:**
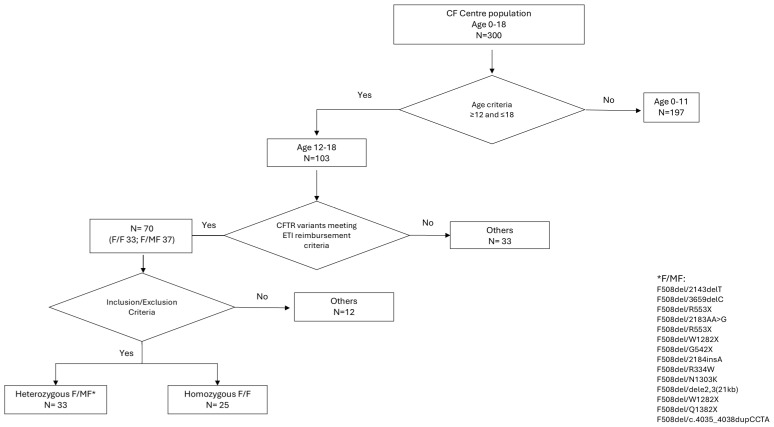
Flow chart.

**Figure 2:**
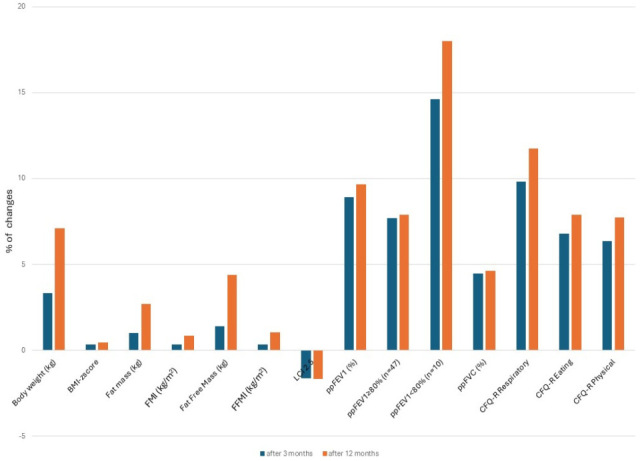
Changes in the mean parameters of nutritional, pulmonary function, and quality of life after 3 and 12 months of treatment with elexacaftor/tezacaftor/ivacaftor (ETI). Abbreviations: BMI, body mass index; FMI, fat mass index; FFMI, fat-free mass index; LCI, lung clearance index; ppFEV_1_, percent predicted FEV_1_; ppFVC, percent predicted FVC; CFQ-R, Cystic Fibrosis Questionnaire-Revised.

From July 2022 to December 2023, data from 58 pwCF (mean age 14.19 ± 1.78; 29 (50%) females; 25 (43%) homozygous F508del) receiving ETI therapy were collected. A flow chart is presented in Figure 1. The baseline characteristics of the study cohort, which met the inclusion criteria, and changes in nutritional status indices, PFT results, and quality-of-life assessments after 3 and 12 months of treatment are shown in Table 2 and Figure 2.

### 3.2. Nutritional Status Assessment

The data presented in Table 2 describe longitudinal changes in anthropometric and body composition parameters assessed at baseline (0 months), 3 months, and 12 months. A statistically significant increase in body weight was observed over time, with a mean gain of 3.33 kg at 3 months and 7.10 kg at 12 months, indicating a sustained effect on body mass.

A significant increase in the BMI z-score) was also noted, with values shifting from −0.90 ± 0.95 at baseline to 0.05 ± 0.82 at 3 months and 0.18 ± 0.89 at 12 months, suggesting a marked normalisation in the overall nutritional status.

Fat mass exhibited a significant increase over time, with mean values rising from 10.88 ± 3.72 kg at baseline to 12.08 ± 4.19 kg at 3 months and 13.64 ± 4.73 kg at 12 months. A concurrent increase in the Fat Mass Index (FMI) was observed, with changes of +0.35 ± 0.79 at 3 months and +0.86 ± 0.88 at 12 months, indicating a proportional increase in fat mass relative to height.

Fat-free mass (FFM) also demonstrated a significant increase, suggesting improvements in lean body mass. The change in FFM (kg) from baseline was +1.41 ± 5.52 at 3 months and +4.40 ± 3.13 at 12 months. Similarly, the Fat-Free Mass Index (FFMI) increased significantly, reflecting a height-adjusted increase in lean mass, with changes of +0.36 ± 2.05 at 3 months and +1.06 ± 0.85 at 12 months.

These changes in nutritional and body composition parameters are visualised as percentage gains in Figure 2. FFM showed a more substantial gain compared to fat mass, suggesting an improvement in lean body mass.

At baseline, 26 participants were classified within the optimal BMI range (50–85th percentile). This number rose to 32 at 3 months but declined to 27 by 12 months (Table 3). The number of participants categorised between the 10th and 24th percentile steadily decreased from 13 at baseline to 5 at 12 months. Similarly, those with lower BMI (below the 10th percentile) declined from 6 at baseline to 3 at 3 months, remaining stable through 12 months.

In contrast, the number of overweight individuals (85–94.9th percentile) increased from 1 at baseline to 10 at 12 months. The obese category (above 95th percentile) was not present at baseline but was observed in 1 participant at 3 months and remained constant at 12 months.

### 3.3. Pulmonary Function Assessment

There were statistically significant differences in lung function parameters such as percent predicted FEV_1_ (ppFEV_1_), percent predicted FVC (ppFVC), and LCI after 3 and 12 months of ETI therapy (Table 2, Figure 2). At the 3-month follow-up, a significant improvement was observed in ppFEV1 (8.91 ± 8.23, 95% CI: 6.94–10.11), ppFVC (4.46 ± 5.24, 95% CI: 4.42–6.43), with a median decrease in LCI (−1.62 ± 2.15, 95%: 1.81–2.64). After 12 months, improvements in pulmonary parameters were maintained: ppFEV1 (9.66 ± 8.77, 95% CI: 7.40–10.76), ppFVC (4.61 ± 5.70, 95% CI: 4.81–6.99), and LCI (−1.68 ± 1.89, 95% CI: 1.58–2.33).

After 3 months of ETI therapy, the mean increase in ppFEV_1_ was significantly greater in patients with a baseline ppFEV_1_ < 80% (*n* = 10, 14.6 ± 6.91) compared to those with a baseline ppFEV_1_ ≥ 80% (*n* = 47, 7.70 ± 8.04, *p* = 0.015). This difference persisted and became more noticeable at 12 months, with the ppFEV_1_ < 80% group showing a mean increase of 18.00 ± 7.87 versus 7.89 ± 7.95 in the ppFEV_1_ ≥ 80% group (*p* < 0.001). Regarding LCI, a trend towards a greater reduction was observed in the ppFEV_1_ < 80% group compared with the ppFEV_1_ ≥ 80% group; however, this difference did not reach statistical significance. The improvement in PFT results was similar across patients, regardless of gender and genotype (F/F and F/MF).

In most adolescents, an improvement in PFT outcomes was observed at the 3-month follow-up. The results showed increases in spirometric parameters: ppFEV_1_ in 45 (82%), ppFVC in 40 (73%), and a decrease in the LCI coefficient in 49 patients (89%). This effect was maintained at 12 months after the start of therapy, with a rise in ppFEV_1_ in 85%, ppFVC in 78%, and a reduction in the LCI coefficient in 87% of individuals.

The analysis also revealed a significant correlation (Spearman’s Rho) between improvements in PFT parameters (ppFEV_1_, ppFVC, and LCI) and changes in specific indices (body mass, BMI, FFM, FFMI) used to assess the patients’ nutritional status, as well as with the CFQ-R Respiratory domain measuring quality of life (Table 4 and Supplementary Materials).

### 3.4. Health-Related Quality of Life Assessment

In the CFQ-R Respiratory domain, scores increased significantly from 81.71 ± 16.79 at baseline to 91.71 ± 11.34 at the 3-month follow-up (mean change), and this improvement was sustained at one year after the initiation of ETI therapy (mean change from baseline, +11.75 ± 22.25). Figure 2 illustrates the changes in CFQ-R parameters over the observational period. An improvement of 4 points is regarded as the minimal clinically important difference; 67% of patients achieved an improvement greater than this threshold at 3 months, and 69% at 12 months.

Results in the CFQ-R Eating domain demonstrated a gradual initial increase, from 83.65 ± 17.78 at baseline to 86.94 ± 15.15 at 3 months, followed by a significant advancement to 91.22 ± 12.01 at 12 months, indicating an improvement in this aspect of quality of life over the long term.

A significant change in scores was also observed over time in the CFQ-R Physical scale. Results showed an improvement of +6.34 ± 17.78 (*p* = 0.003) after 3 months, and +7.74 ± 13.69 (*p* < 0.001) at the one-year follow-up. Detailed results for all three CFQ-R domains, along with changes observed during the first year of treatment, are presented in Table 2.

### 3.5. Assessment of the Relationship Between Pulmonary Function, Nutritional Status, and Quality of Life

#### 3.5.1. Pulmonary Function and Nutritional Status

Analysis of the association between parameter changes over two observation periods—3 and 12 months—revealed significant positive correlations during the first 3 months between changes in spirometric parameters (ppFEV_1_ and ppFVC) and changes in body weight (0.425, 0.488) and BMI z-scores (0.443, 0.407). After 12 months, the relationships between changes in spirometric parameters and the analysed nutritional indices (body weight: 0.353, 0.398 and BMI z-score: 0.417, 0.468) remained statistically significant. However, their strength was lower compared to the 3-month period (Table 4 and Supplementary Materials).

Analysis of the MBNW parameter (LCI) and BMI z-score revealed a significant negative correlation across two observation periods (−0.280 and −0.341, respectively).

A similar level of correlation was observed between FFM (kg) and FFMI (kg/m^2^) with ppFEV_1_ and ppFVC values, both in the 3-month (0.423; 0.419; 0.382; 0.376) and 12-month periods (0.33; 0.275; 0.357;0.380). However, FM (kg) and FMI (kg/m^2^) did not show significant correlations with changes in ppFEV_1_. Nonetheless, a significant relationship was found with ppFVC in both observation periods (0.298; 0.290; 0.321; 0.292) (Table 4 and Supplementary Materials).

#### 3.5.2. Pulmonary Function and Quality of Life

An assessment of changes in pulmonary symptoms after the first 3 months of ETI therapy revealed a strong correlation between the CFQ-R Respiratory scale and PFT results (ppFEV_1_: 0.439; ppFVC: 0.464). This significant relationship persisted at the 12-month follow-up (ppFEV_1_: 0.472; ppFVC: 0.537). Additionally, a negative correlation was found between changes in LCI and CFQ-R Respiratory responses after 3 months (−0.284). There were no statistically significant relationships between changes in the CFQ-R Physical and CFQ-R Eating domains and PFT results or the MBNW parameter (LCI).

#### 3.5.3. Nutritional Status and Quality of Life

The analysis of the association between nutritional status and HRQoL parameters revealed a strong correlation between changes in the CFQ-R Respiratory Scale and BMI z-score at 3 months, which remained significant at 12 months (0.470 and 0.406, respectively).

A similar correlation between changes in the CFQ-R Respiratory scale and Body weight (0.335), FFM (kg) (0.379), and FFMI (kg/m^2^) (0.375) was observed only during the first 3 months (Table 4 and Supplementary Materials).

## 4. Discussion

The study fills an important evidence gap by providing real-world paediatric data from a modulator-naïve cohort. Conducted in a national context where ETI therapy has only recently become reimbursed, it demonstrates sustained improvements in lung function, nutritional status, and quality of life during the first year of treatment. The integrated analysis of these parameters underscores the systemic benefits of HEMT and its impact on the early stages of CF, extending current knowledge from clinical trials.

The marked improvement observed in the present cohort may be related to the specific characteristics of the studied group. The analysis was conducted within a national framework in which ETI therapy is reimbursed for patients carrying specific CFTR variants, as clearly delineated in the national treatment programme (Figure 1). The population included only adolescents aged 12–17 years, excluding adult patients, in whom most disease-related changes are not yet irreversible, allowing for a stronger therapeutic response. Baseline clinical condition and population-specific factors, including environmental and healthcare-related aspects of the Warsaw CF cohort, may also have contributed to the favourable outcomes.

The introduction of mCFTR in the last decade has been a breakthrough in CF management, transforming the landscape of clinical care [33]. They alter the course of the disease, resulting in a longer lifespan for patients and a better quality of life. Many clinical studies have confirmed their effect on improving clinical condition, including nutritional health, maintaining normal lung function, slowing disease progression, and decreasing the frequency of PEx.

The latest systematic review stresses that clinical and observational studies have demonstrated that the combination therapy ETI exerts a significant beneficial effect on various clinical parameters in pwCF. A substantial improvement in ppFEV1 was observed in both adults and children. Additionally, an improvement in LCI was noted. ETI therapy also contributed to an increase in body weight and body mass index (BMI). Studies have shown a significant rise in BMI and body weight among patients of different ages and genotypes following the initiation of treatment. This effect may result from improved appetite, increased food intake, and potential enhancement of pancreatic exocrine function, leading to more efficient intestinal nutrient absorption. A significant improvement was also recorded in quality of life, as assessed by the CFQ-R RD [12].

In the context of transformative changes in CF treatment, which have led to a slowdown in the progression of pulmonary disease, particularly in the paediatric population, a comprehensive assessment of the effectiveness of basic defect treatment in clinical practice is essential.

### 4.1. Nutritional Aspects

The impact of nutritional status on the survival of pwCF is well-documented, and nutritional therapy is a significant component of treating this disease. Malnutrition has a negative effect on lung function, as well as on the quality and length of life [16]. Over the past twenty years, there has been a significant decrease in the prevalence of malnutrition among cystic fibrosis patients, with a simultaneous increase in cases of overweight and obesity [34].

BMI as the sole parameter of nutritional status is insufficient. Lean body mass content is a more sensitive prognostic indicator of lung function than BMI. Decreased lean body mass and/or excess fat tissue are often observed in cystic fibrosis patients who have a normal BMI value. Current European nutritional guidelines recommend including body composition analysis in assessing nutritional status. One of the recommended tools for this purpose is bioelectrical impedance analysis (BIA), which enables the determination of individual components such as fat tissue and lean body mass. BIA is also a safe and non-invasive method [15].

Improving nutritional status, supported by VST, underscores the importance of monitoring both anthropometric parameters and body composition in preventing overweight and obesity.

Data on changes in body composition in the paediatric population treated with ETI are limited.

Our data demonstrates a statistically significant and sustained improvement in anthropometric and body composition parameters over a 12-month period. Our findings indicate not only absolute weight gain but also an improvement in nutritional status relative to age- and sex-adjusted norms. These findings align with earlier studies demonstrating improved nutritional status following ETI treatment in paediatrics [35].

Although data on body composition changes in paediatric patients receiving ETI are limited, our findings demonstrate similarly robust improvements. FM and FFM both increased significantly over time. Importantly, these gains were reflected in height-adjusted indices: FMI and FFMI increased significantly, underscoring improvements in both adipose and lean tissue compartments independent of growth in stature. The concurrent increase in FFMI suggests a positive effect on muscle mass, a clinically relevant outcome in CF, where muscle depletion is associated with poorer clinical trajectories. These findings are consistent with reports from López Cárdenes et al., who also observed significant increases in FM and FFM over 12 months in children with CF treated with ETI [35]. In contrast, the study by Boat et al. found no significant association between changes in body composition and FEV_1_, and noted that improvements in FMI, FFMI, and Skeletal Muscle Mass Index (SMMI) did not translate into changes in z-scores [36].

The pattern of BMI change after ETI initiation suggests that while early nutritional recovery is achievable, sustaining these benefits requires ongoing clinical attention. Strategies should aim not only to promote healthy weight gain but also to mitigate the potential for excessive weight gain and obesity as treatment continues. In the study by Enaud et al., 7 of 62 patients became overweight after one year of treatment [37]. Similarly, in our cohort, the number of overweight participants (85–94.9th percentile) increased significantly from 1 at baseline to 10 at 12 months. Additionally, one participant progressed to obesity (>95th percentile) after 12 months of ETI treatment.

Based on our results, the data strongly suggest that the observed increase in BMI and fat mass represents both “catch-up growth” for a previously undernourished cohort and an emerging long-term risk of overweight/obesity for a new subset of patients.

This trend highlights the importance of monitoring both undernutrition and the emerging risk of overweight in paediatric CF patients on ETI. However, in our cohort, the percentage change reflecting the increase in FFM was substantially higher than the increase observed in FM.

### 4.2. Pulmonary Aspects

In our study, three months after starting ETI, we observed a significant improvement in pulmonary parameters (ppFEV_1_, ppFVC, LCI), which was maintained throughout the one-year observation period.

The findings regarding FEV_1_ follow the data from the phase 3 clinical trials of ETI [3,38] as well as other real-world studies of similar age groups reporting outcomes over 12 months [9,13,14,39].

The clinical efficacy of ETI was demonstrated by Connett et al. in a cohort of adolescents with CF (*n* = 62, mean age 13.3 years) within the first two years of its availability at a single CF centre in the UK. Consistent with our findings, there was a significant increase in mean FEV_1_% of 11.7 units (95% CI 7.4–15.6) observed from baseline within four months of starting ETI, and this improvement was maintained throughout the two-year treatment period [39]. Improvements were similar across all treatable genotypes (76% were homozygous for F508del) and in patients who received mCFTR therapy before initiating ETI treatment (*n* = 41, 66%) [39].

The delay in the availability of mCFTR in Poland resulted in all patients in our study being mCFTR-naïve.

Enhancement in lung function, respiratory symptoms, and BMI was also found in an extensive real-world observational study, PROMISE, in a diverse US population who were starting ETI therapy for the first time [13]. The study demonstrated the extensive effects of ETI over 30 months of clinical use in 487 pwCF aged 12 years or older (average age was 25.1 years), all of whom had at least one F508del allele (48.5% were F508del homozygous, and 40% were heterozygous F508del and MF). After six months of starting ETI, ppFEV_1_ increased by 9.76 points (95% CI, 8.76 to 10.76) and the CFQ-R respiratory score by 20.4 points (95% CI, 18.3 to 22.5). Larger improvements (10.8; 95% CI, 9.3 to 12.4) were noted in those who were naïve to mCFTR (50.9%) [13].

Another extensive observational cohort study using data from the German CF Registry, involving 67 centres and including 2645 adolescents (12 to <18 years, *n* = 614, 23.2%), showed improvements in ppFEV_1_ over the first year after ETI initiation (11.3%, 95% CI, 10.8–11.8, *p* < 0.0001). The most significant increases were observed in individuals who had not previously undergone mCFTR therapy [14].

Additionally, a retrospective analysis of records from the German CF paediatric population is also available [9]. The study included two groups of children: 6–11-year-olds (*n* = 22) and 12–17-year-olds (*n* = 24). Clinical outcomes, spanning PFTs, were assessed at baseline, 3 months, and 6 months after the start of ETI. Most patients (59%) were homozygous for F508del (F/F), and half of them (50%) were transitioned from another mCFTR. At the 3-month follow-up, ppFEV_1_ increased by 11.4% (95% CI: 8.0–14.9, *p* < 0.0001), with no further significant change after 6 months. No significant differences were observed between the age groups. This study found that PFTs showed greater benefits for children with the F/MF genotype compared to those with the F/F genotype. The positive effect observed after three months of ETI therapy persisted at the six-month follow-up [9].

Similarly to Olivier et al. [9], we also observed that although the average ppFEV_1_ in our patients was normal at baseline, a subgroup of children with impaired pulmonary function (ppFEV_1_ < 80%) experienced the greatest increase in ppFEV_1_ compared to those with normal lung function (ppFEV_1_ ≥ 80%) after three months, and this effect was more pronounced at twelve months. Our findings support early treatment with ETI, particularly in patients with impaired lung function. Additionally, we noted a trend towards a greater reduction in LCI within this subgroup. It proves the specific therapeutic benefit of ETI for these patients.

Our study also demonstrated that improvements in spirometric parameters (ppFEV_1_, ppFVC) were supported by rapid and sustained increases in LCI. This marker, derived from MBNW, measures ventilation heterogeneity and is most sensitive for early detection and monitoring progression in CF lung disease. While ppFEV_1_ may exhibit ceiling effects in individuals with preserved lung function, LCI offers a more sensitive assessment of ventilation distribution and peripheral airway involvement. It therefore enables earlier detection of treatment responses, particularly in patients with mild disease.

The presented outcomes confirm that LCI is a more sensitive indicator of lung function changes in CF compared to spirometry, especially during childhood. It is very useful for monitoring early lung disease and assessing therapeutic efficacy.

These findings align with the literature reporting a significant improvement in LCI with ETI therapy (35–37). Zemanick et al., based on a 24-week open-label phase 3 study, reported a decrease in LCI (−1.71; 95% CI, −2.11 to −1.30) in children aged 6 to 11 years with at least one F508del allele [40].

Similar results showing a meaningful reduction in LCI were observed in younger children aged 2–5 years after 6 months of ETI treatment. Decreases in LCI (−0.83 U; 95% CI, −1.01 to −0.66; *n* = 50) were detected from baseline through 24 weeks [41].

Along with MBNW, lung MRI (magnetic resonance imaging) is also a promising and sensitive method that enables monitoring of CF lung disease, especially in its early stages, and assessing the effects of treatment, including mCFTR. Unfortunately, we do not yet have the capacity to perform an MRI at our centre. However, we hope that we will be able to perform it both during research and in clinical practice in the near future.

Graeber et al.’s data demonstrated improvements in lung ventilation and abnormalities in lung morphology, based on MBNW and MRI, in adolescent and adult patients with CF and one or two F508del alleles in a real-world setting. Treatment with ETI reduced LCI in F508del/MF (−2.4; interquartile range [IQR], −3.7 to −1.1; *p* < 0.001) and F508del homozygous (−1.4; IQR, −2.4 to −0.4; *p* < 0.001) patients. Additionally, ETI improved the MRI global score [42].

Streibel et al. also reported an improvement in LCI and MRI-derived ventilation and perfusion measures after a mean follow-up of 4 months after the start of ETI therapy. In 24 children with CF, with a mean age of 13.8 years (8.6 to 17.2), eleven patients with the F508del/F508del genotype and 13 with the F508del/MF genotype showed a significant improvement in lung function (LCI −0.84 [−1.62 to −0.06]; FEV1 [z-score] 1.05 [0.56 to 1.55]) and in structural and functional MRI parameters [43].

Stahl et al. conducted a prospective, observational, multicentre, post-approval study to examine the effect of ETI on the LCI and lung MRI score in children with CF aged 6–11 years (*n* = 107). The study included 40 participants who were heterozygous for F508del and a minimal function variant (F/MF), and 67 who were homozygous for F508del (F/F). Treatment with ETI improved the median (interquartile range (IQR)) LCI in children with F/MF (−1.0 (−2.0–−0.1); *p* < 0.001) and F/F (−0.8 (−1.9–−0.2); *p* < 0.001) [44].

Research from both real-world data and randomised clinical trials supports our findings, endorsing the use of ETI as a highly effective treatment option for individuals with CF who have at least one F508del allele. To monitor early changes in lung function, LCI appears to be the most sensitive marker for detecting alterations after commencing ETI.

### 4.3. Psychological Aspects

The introduction of CFTR modulator therapy has been long-awaited by individuals with cystic fibrosis and their caregivers. The implementation of this therapy is associated with immense expectations and hope for significant improvements in health, physical functioning, as well as psychological well-being and quality of life. Observations from countries where this treatment has already been introduced indicate that the vast majority of patients experience both physical and psychological benefits. However, it may also have indirect negative effects on the well-being of pwCF and their caregivers, who frequently report difficulties with adjustment [1].

For the assessment of patient-reported outcomes (PROs) related to the efficacy of ETI, HRQoL measured with the CFQ-R has served as an important endpoint in numerous randomised controlled trials and observational studies, with the CFQ-R Respiratory Scale being the most frequently applied domain [3,6,12,13,38,40,45,46].

At baseline, our patients aged 12–18 years achieved relatively high scores in the CFQ-R Respiratory domain (mean 81.71), indicating that even before initiation of ETI therapy, they perceived their HRQoL in this area as good. Nevertheless, after one year of treatment, the mean score increased by 11.75 points. For comparison, in the study by Zemanick [40], children aged 6–11 years had a baseline Respiratory domain score of 80.3, with an improvement of 7.0 points after 24 weeks of therapy. In contrast, a study with adults and adolescents > 12 years of age (mean age ∼ 26 years) demonstrated lower baseline scores (68.3) but a greater increase after 24 weeks of ETI treatment, with a mean improvement of 17.5 points [3]. Moreover, adult patients with a baseline ppFEV_1_ of ∼30% achieved a 32.6-point increase in the CFQ-R Respiratory domain after one year of ETI therapy, improving from a mean of 55.5 at baseline to 94.4 at week 48 [47]. These findings suggest that older patients, who generally present with more advanced lung disease, tend to report lower baseline HRQoL in the CFQ-R Respiratory domain but demonstrate larger improvements following ETI initiation.

Several studies have analysed all CFQ-R domains rather than focusing solely on the Respiratory domain, demonstrating improvements in most areas within a few months of initiating ETI therapy [48,49,50]. These findings are in line with our results, which showed notable improvement after 3 months in the Physical Functioning and Eating Problems domains, with effects sustained at one-year follow-up. Importantly, the patient-reported improvements in health-related quality of life observed in the questionnaires were also reflected in real-world experiences. Many patients reported a noticeable enhancement in their physical condition, with several beginning to engage in sports activities, cycling, or other forms of exercise. This general improvement in health also led to meaningful changes in patients’ lifestyles and how they perceived their own physical capabilities and potential. The substantial improvement observed in patients receiving ETI therapy may, in part, be attributed to the long-standing anticipation surrounding the treatment. Patients, caregivers, and healthcare providers held high expectations regarding its efficacy and its potential impact on quality of life.

However, there is also the risk of adverse psychological effects, including symptoms of depression and anxiety, as well as thoughts and attempts of suicide. Even before the introduction of mCFTR therapy, pwCF had a 2–3 times greater risk of experiencing symptoms of depression and anxiety compared to the general population. Depression and anxiety, in turn, are associated with deterioration in health status, mainly resulting from non-adherence to therapeutic recommendations. Therefore, it is extremely important to assess their functioning and implement appropriate interventions in case of troubling symptoms during mCFTR therapy [1,18,48,49,50].

### 4.4. Correlations Between Lung Function, Nutritional Status, and Psychological Well-Being

Our findings indicate that the most pronounced associations between lung function and nutritional status occurred during the first 3 months of therapy. Short-term improvements in body weight, BMI z-score, and FFM were strongly correlated with gains in spirometric parameters (ppFEV_1_, ppFVC), suggesting a close link between early nutritional recovery and pulmonary function. Similarly, during this period, changes in the CFQ-R Respiratory domain showed strong correlations with both nutritional and lung function indicators. Comparing these findings with the second period (12 m vs. 0 m), we confirmed that the improvements in key nutritional, pulmonary, and psychological parameters achieved at the start of therapy were maintained over the first year.

### 4.5. Strengths and Limitations

The main strength of this study is the comprehensive evaluation of ETI therapy’s effectiveness in a relatively large group of patients who are naïve to mCFTR, all treated at a single CF centre. We combine clinical assessment, nutritional status, and PFT results, including MBNW, with quality of life in the paediatric group, enabling an in-depth analysis of the treatment response in clinical practice. Conversely, the fact that this was a single-centre study may limit the generalisability of our results to a broader population of pwCF. The open-label, single-arm design without a comparator group limits the interpretation of the findings and the ability to assess the relative efficacy and safety of the intervention directly. Furthermore, the 12-month follow-up period might not have been sufficient to fully record the effects of ETI over time. Additional research is required to investigate long-term outcomes.

Another limitation is the potential for selection bias, for example, the exclusion of patients unable to perform PFTs. In turn, the lack of a control/comparator group limits causal inference.

The study did not include a systematic assessment of sinonasal function or upper airway symptoms, and data on chronic rhinosinusitis were not collected. This limits the ability to evaluate the potential effects of ETI on upper airway outcomes.

Furthermore, our centre’s inability to perform MRI has restricted our ability to evaluate morphology and has hindered more precise assessment of lung ventilation in early CF lung disease.

The limitations also include the retrospective nature of the study, as well as the age and genotype of the patients involved, which are determined by the mCFTR reimbursement requirements in Poland. A future prospective study with younger children using MRI is planned, following the mCFTR registration guidelines.

## 5. Conclusions

In summary, the clinical results of this retrospective observational study demonstrate a strong health benefit of ETI in real-world practice, consistent with those observed in controlled clinical trials and other observational studies. This single-centre study revealed comprehensive clinical advantages of this therapy, leading to significant and enduring improvements in nutritional status, body composition, pulmonary function, and quality of life among adolescents with CF throughout one year, particularly in patients with advanced disease.

Alongside mCFTR, increasingly used in pwCF, new challenges are emerging, such as monitoring treatment and finding more sensitive parameters to evaluate effectiveness, especially in early CF lung disease in children. Preventing and treating conditions that were previously less common or did not occur at all, like obesity or depression, is also equally important. This study emphasises the importance of multidisciplinary follow-up, involving pulmonologists, nutritionists, and psychologists to monitor clinical outcomes, nutritional status, and quality of life. Long-term longitudinal research extending beyond 24 months and incorporating MRI-based markers is necessary to further evaluate the systemic and pulmonary effects of therapy and enhance patient care.

## Figures and Tables

**Table 1:** Inclusion and exclusion criteria for the study group. Abbreviations: CF, cystic fibrosis; ETI, elexacaftor/tezacaftor/ivacaftor; PFTs, pulmonary function tests.

Inclusion Criteria	Exclusion Criteria
confirmed diagnosis of CF based on current diagnostic criteria [18,20,21,22] patient’s age between 12 and 17 years qualification for ETI therapy according to the criteria of the National Health Fund of Poland, based on the patient’s age and genotype ability to undergo PFTs no history of transplantation (any organ) stable clinical condition informed consent from the patient and their caregiver to participate in the retrospective observational study	patient’s age below 12 years or above 17 years lack of consent from the patient and caregiver for ETI therapy lack of cooperation or clinical conditions (e.g., haemoptysis) that prevent the patient from undergoing PFTs lack of informed consent from the patient and their caregiver to participate in the retrospective observational study

**Table 2:** Group characteristics at baseline and changes in indices of nutritional status, results of pulmonary function tests, and assessment of quality of life after 3 and 12 months of treatment with elexacaftor/tezacaftor/ivacaftor (ETI). *Abbreviations: BMI, body mass index; FMI, Fay Mass Index; LCI, lung clearance index; ppFEV_1_, percent predicted FEV_1_; ppFVC, percent predicted FVC; CFQ-R, Cystic Fibrosis Questionnaire-Revised*.

Parameters	Points of Assessment									
	Baseline (0 Month)		3 Months		12 Months		Change Between 3 m and 0	*p* *	Change Between 12 m and 0	*p* *
	Mean ± sd	Min–Max	Mean ± sd	Min–Max	Mean ± sd	Min–Max	Mean ± sd		Mean ± sd	
**Age (years)**	14.19 ± 1.78	12–17					n/a	n/a	n/a	n/a
**Body weight (kg)**	52.34 ± 11.64	32.3–90.3	55.66 ± 11.56	36.7–96.8	59.40 ± 11.96	39.40–106.3	3.33 ± 2.30	<0.001	7.10 ± 4.62	<0.001
**Body weight (kg)** **Female/Male**	49.82 ± 6.28/54.86 ± 14.89		52.87 ± 5.60/58.48 ± 14.94		55.56 ± 6.24/63.37 ± 14.67					
**BMI-zscore**	−0.30 ± 0.95	−3.39–1.72	0.05 ± 0.82	−2.33–1.83	0.18 ± 0.89	−2.59–1.96	0.35 ± 0.36	<0.001	0.47 ± 0.46	<0.001
**BMI-zscore** **Female/Male**	−0.08 ± 0.73/−0.51 ± 1.09		0.23 ± 0.60/−0.12 ± 0.96		0.29 ± 0.77/0.06 ± 1.02					
**Fat mass (kg)**	10.88 ± 3.72	5.30–21.6	12.08 ± 4.19	5.10–25.7	13.64 ± 4.73	5.00–29.20	1.02 ± 2.09	<0.001	2.70 ± 2.51	<0.001
**Fat mass (kg)** **Female/Male**	12.46 ± 2.90/9.3 ± 3.3		13.89 ± 3.15/10.4 ± 4.27		15.37 ± 3.69/11.85 ± 4.93					
**FMI (kg/m^2^)**	4.02 ± 1.32	1.80–7.31	4.43 ± 1.54	1.89–8.33	4.90 ± 1.75	1.85–9.49	0.35 ± 0.79	<0.001	0.86 ± 0.88	<0.001
**FMI (kg/m^2^)** **Female/Male**	4.84 ± 1.06/3.22 ± 0.98		5.37 ± 1.24/3.57 ± 1.19		5.85 ± 1.50/3.91 ± 1.38					
**Fat Free Mass (kg)**	41.47 ± 9.75	25–68.7	43.66 ± 9.66	28.6–71.10	45.76 ± 9.86	29.70–77.10	1.41 ± 5.52	<0.001	4.40 ± 3.13	<0.001
**Fat Free Mass (kg)** **Female/Male**	37.36 ± 3.96/45.49 ± 11.92		39.06 ± 3.21/47.99 ± 11.59		40.20 ± 3.48/51.51 ± 10.81					
**FFMI (kg/m^2^)**	15.13 ± 1.92	11.41–20.51	15.77 ± 1.82	12.71–21.00	16.16 ± 1.99	12.85–22.28	0.36 ± 2.05	<0.001	1.06 + 0.85	<0.001
**FFMI (kg/m^2^)** **Female/Male**	14.52 ± 1.23/15.69 ± 2.27		15.07 ± 0.95/16.40 ± 2.16		15.26 ± 1.17/17.09 ± 2.21					
**LCI_2.5_**	9.52 ± 2.62	6.08–16.14	7.71 ± 1.91	6.08–14.88	7.81 ± 2.35	6.00–20.39	−1.62 ± 2.15	<0.001	−1.68 ± 1.89	<0.001
**LCI_2.5_ Female/Male**	8.96 ± 2.70/10.04 ± 2.45		7.78 ± 2.1/7.62 ± 1.66		7.77 ± 2.91/7.82 ± 1.61					
**ppFEV_1_ (%)**	92.09 ± 17.67	38.00–134.0	100.91 ± 14.9	51.00–130.00	101.65 ± 14.92	50.00–136.00	8.91 ± 8.23	<0.001	9.67 ± 8.77	<0.001
**ppFEV_1_ (%) Female/Male**	95.39 ± 17.69/89.43 ± 16.8		101.52 ± 14.71/100.75 ± 14.95		102.89 ± 14.82/100.82 ± 14.6					
**ppFEV_1_ ≥ 80% (*n* = 47)**	97.83 ± 12.11	81.00–134.00	105.53 ± 10.05	85.00–130.00	105.72 ± 11.23	77.00–136.00	7.70 ± 8.04	<0.001	7.89 ± 7.95	<0.001
**ppFEV_1_ < 80% (*n* = 10)**	65.10 ± 14.53	38.00–78.00	79.70 ± 16.78	51.00–99.00	83.10 ± 17.10	50.00–105.00	14.60 ± 6.91	0.004	18.00 ± 7.89	0.004
**ppFVC (%)**	98.31 ± 15.39	42.00–134.00	102.88 ± 13.29	48.00–131.00	103.00 ± 12.88	49.00–135.00	4.46 ± 5.24	<0001	4.61 ± 5.70	<0.001
**ppFVC (%) Female/Male**	100.11 ± 15.0/97.25 ± 15.11		104.07 ± 13.07/102.11 ± 13.27		104.86 ± 12.80/101.57 ± 12.47					
**CFQ-R Respiratory**	81.71 ± 16.79	22.22–100.00	91.71 ± 11.43	50.00–100.00	93.85 ± 11.18	33.33–100.00	9.83 ± 16.48 **	<0.001	11.75 ± 15.62 **	<0.001
**CFQ-R Respiratory** **Female/Male**	83.13 ± 19.03/81.13 ± 12.55		93.01 ± 7.53/91.05 ± 13.93		94.72 ± 8.67/92.96 ± 13.16					
**CFQ-R Eating**	83.65 ± 17.78	33.33–100.00	86.94 ± 15.16	33.33–100.00	91.22 ± 12.01	55.55–100.00	6.79 ± 22.58	0.088	7.90 ± 17.37	0.001
**CFQ-R Eating** **Female/Male**	81.75 ± 18.59/86.57 ± 16.03		85.44 ± 1.04/89.30 ± 11.90		92.06 ± 10.22/91.2 ± 12.72					
**CFQ-R Physical**	88.23 ± 15.70	20.83–100.00	92.81 ± 11.01	58.33–100.00	96.32 ± 7.48	70.83–100.00	6.34 ± 17.78	0.003	7.74 ± 13.69	<0.001
**CFQ-R** **Physical** **Female/Male**	85.3 ± 1.85/91.26 ± 11.87		91.6 ± 10.99/94.70 ± 10.24		94.34 ± 9.20/98.46 ± 4.24					

* Wilcoxon signed-rank test. ** Minimal Clinically Important Difference for CFQ-R Respiratory domain is 4 points [32].

**Table 3:** Category of body mass index (BMI) percentiles over one year of elexacaftor/tezacaftor/ivacaftor therapy.

BMI (Percentiles)	Baseline (n)	3 Months (n)	12 Months (n)
50–85th	26	32	27
25–49th	12	10	11
24–10th	13	9	5
below 10th	6	3	3
85–94.9th	1	3	10
above 95th	0	1	1

0–3 months: χ^2^ = 131.10; χ^2^ (NW) = 82.58; *p* < 0.001; Cramér’s V = 0.758; 3–12 months: χ^2^ (NW) = 71.42; *p* < 0.001; Cramér’s V = 0.686; 0–12 months: χ^2^ (NW) = 57.56; *p* < 0.001; Cramér’s V = 0.685.

**Table 4:** Correlation (Spearman’s Rho coefficient) with changes in nutritional status indices, pulmonary function parameters, and quality of life scores between assessment points (3 months vs. baseline and 12 months vs. baseline). Abbreviations: BMI, body mass index; FMI, Fat Mass Index; FFMI, Fat Free Mass Index; LCI, lung clearance index; ppFEV_1_, percent predicted FEV_1_; ppFVC, percent predicted FVC; CFQ-R, Cystic Fibrosis Questionnaire-Revised.

∆ of Parameters	ppFEV_1_ (%) *		ppFVC (%) *		LCI2.5 *		CFQ-R Respiratory *	
	3 m vs. 0 m	12 m vs. 0 m	3 m vs. 0 m	12 m vs. 0 m	3 m vs. 0 m	12 m vs. 0 m	3 m vs. 0 m	12 m vs. 0 m
**Body weight (kg)**	0.425	0.353	0.488	0.398	-	-	0.335	-
**BMI-zscore**	0.443	0.417	0.407	0.468	−0.280	−0.341	0.470	0.406
**Fat mass (kg)**	-	-	0.298	0.321	-	-	-	-
**FMI (kg/m^2^)**	-	-	0.290	0.292	-	-	-	-
**Fat Free Mass (kg)**	0.423	0.333	0.382	0.357	-	-	0.379	-
**FFMI (kg/m^2^)**	0.419	0.275	0.376	0.380	-	-	0.375	-
**CFQ-R** **Respiratory**	0.439	0.472	0.464	0.537	−0.284	-	n/a	n/a

* The table presents the values of Spearman’s rho coefficient that reached statistical significance (*p* < 0.05). n/a—not applicable.

## Data Availability

The datasets used and analysed during the current study are available from the corresponding author on reasonable request.

## Supplementary Materials

The following supporting information can be downloaded at: https://www.mdpi.com/article/10.3390/jcm14227969/s1.
