# Supplementary material for: Comprehensive Evaluation of Elexacaftor/Tezacaftor/Ivacaftor in Paediatric Cystic Fibrosis: Nutritional, Pulmonary, and Quality-of-Life Outcomes
Source: J Clin Med. 2025 Nov 10;14(22):7969. doi: 10.3390/jcm14227969 (PMC12653656; doi:10.3390/jcm14227969)
Supplement: Supplementary file 1 [file jcm-14-07969-s001.zip › jcm-3909591-supplementary.pdf]

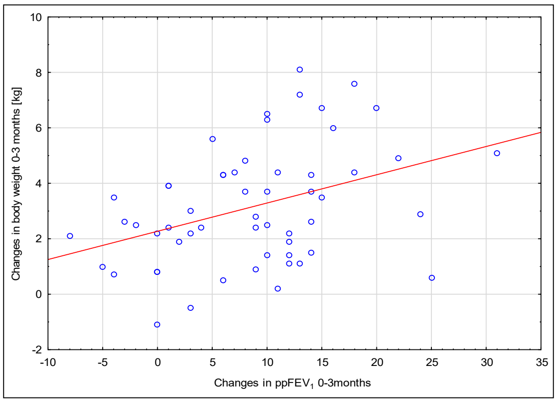

Figure S1. Scatter plot of change in body weight [kg] versus change in ppFEV$_1$ from baseline to 3 months.

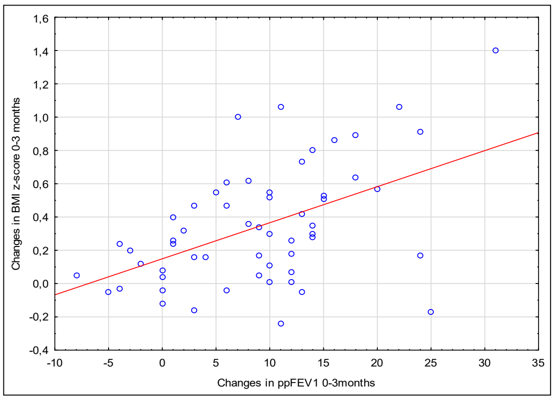

Figure S2. Scatter plot of change in BMI [kg/m^2] versus change in ppFEV$_1$ from baseline to 3 months.

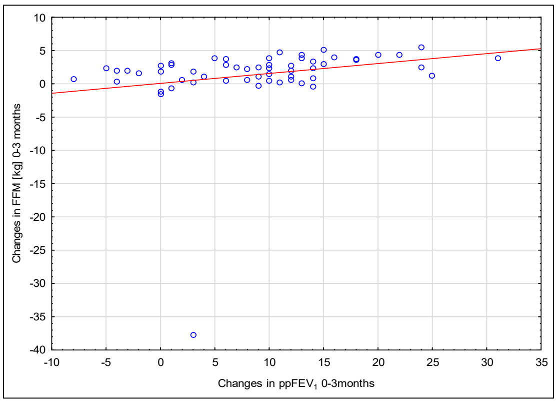

Figure S3. Scatter plot of change in FFM [kg] versus change in ppFEV$_1$ from baseline to 3 months.

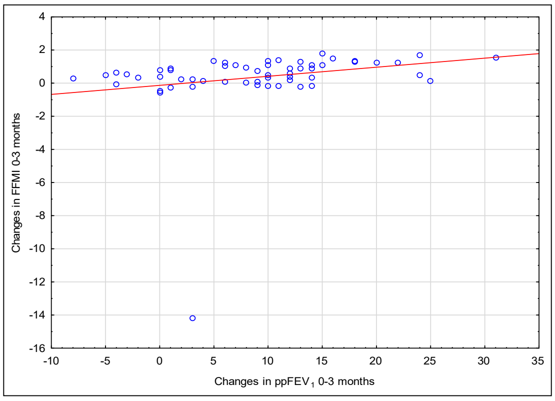

Figure S4. Scatter plot of change in FFMI [kg/m^2] versus change in ppFEV$_1$ from baseline to 3 months.

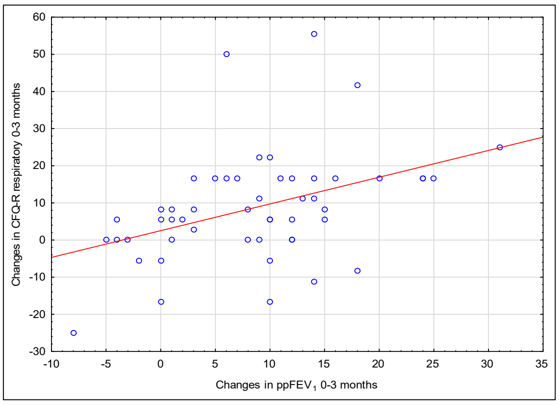

Figure S5. Scatter plot of change in CFQ-R Respiratory versus change in ppFEV$_1$ from baseline to 3 months.

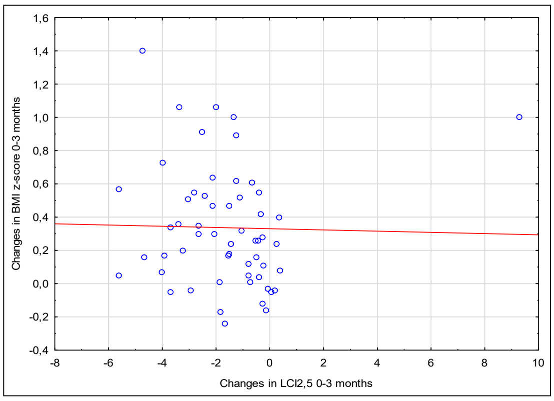

Figure S6. Scatter plot of change in BMIz-score versus change in ppFEV$_1$ from baseline to 3 months.

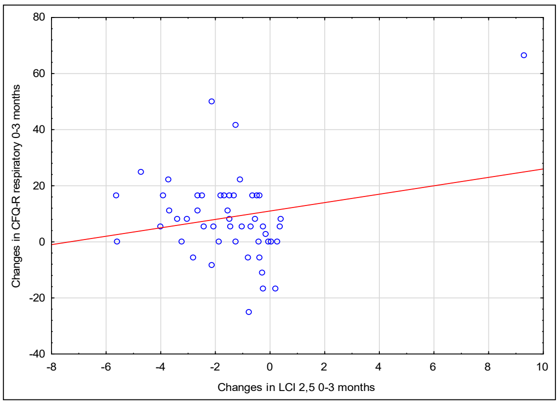

Figure S7. Scatter plot of change in CFQ-R Respiratory versus change in LCI 2,5 from baseline to 3 months.

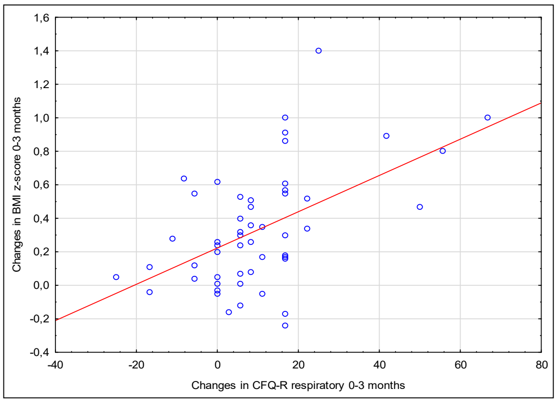

Figure S8. Scatter plot of change in BMI z-score versus change in CFQ-R Respiratory from baseline to 3 months.

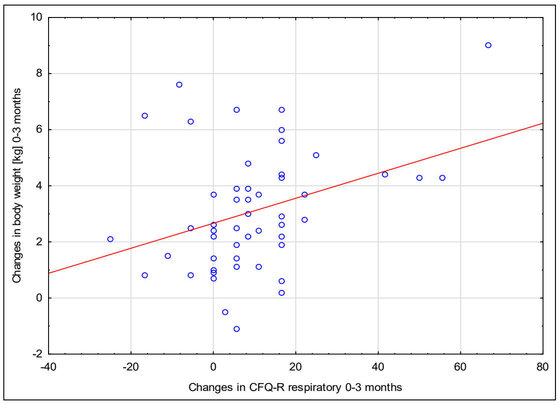

Figure S9. Scatter plot of change inbody weight [kg] versus change in CFQ-R Respiratory from baseline to 3 months.

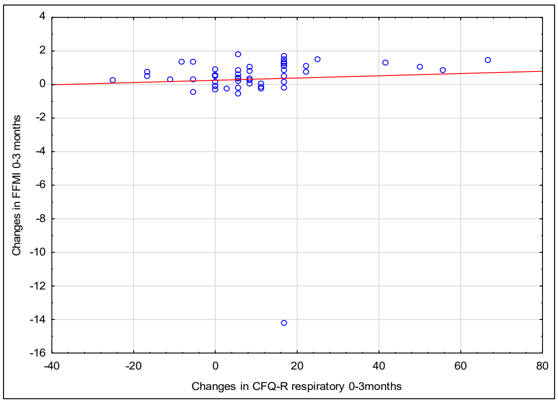

Figure S10. Scatter plot of change in FFMI[kg/m^2] versus change in CFQ-R Respiratory from baseline to 3 months.

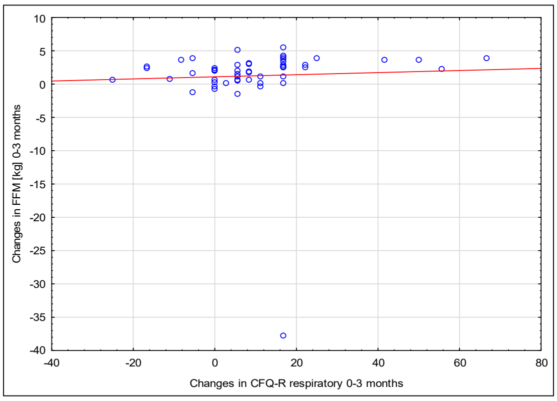

Figure S11. Scatter plot of change in FFM[kg] versus change in CFQ-R Respiratory from baseline to 3 months.

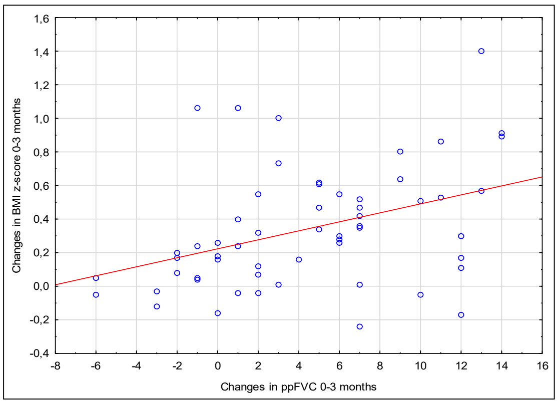

Figure S12. Scatter plot of change in BMI z-score versus change in ppFVC from baseline to 3 months.

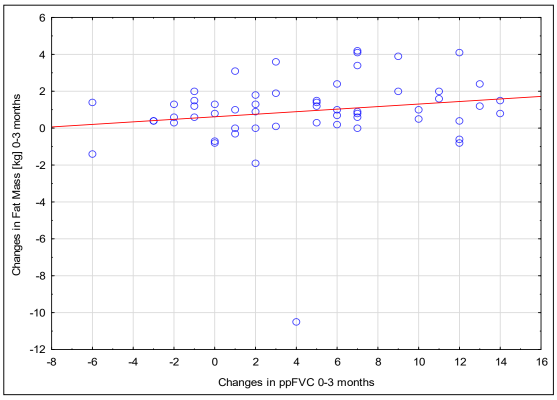

Figure S13. Scatter plot of change in Fat Mass [kg] versus change in ppFVC from baseline to 3 months.

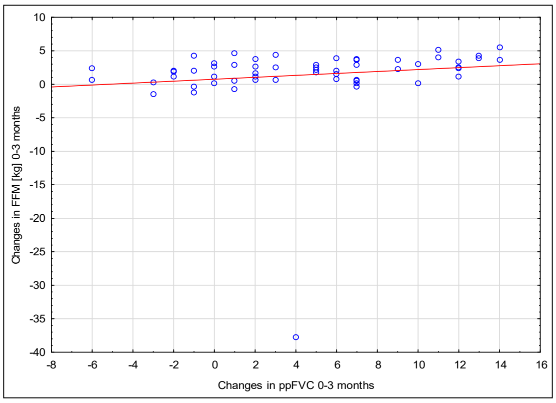

Figure S14. Scatter plot of change in FFM [kg] versus change in ppFVC from baseline to 3 months.

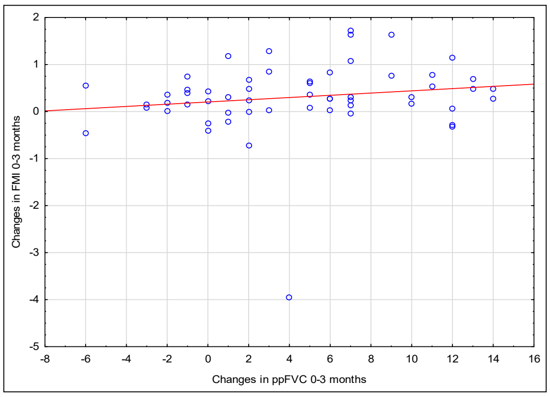

Figure S15. Scatter plot of change inFMI [kg/m^2] versus change in ppFVC from baseline to 3 months.

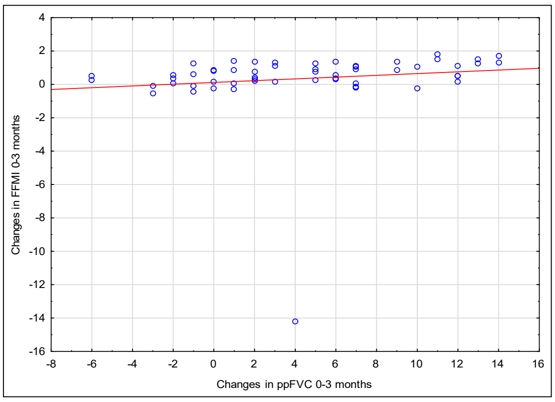

Figure S16. Scatter plot of change in FFMI [kg/m^2] versus change in ppFVC from baseline to 3 months.

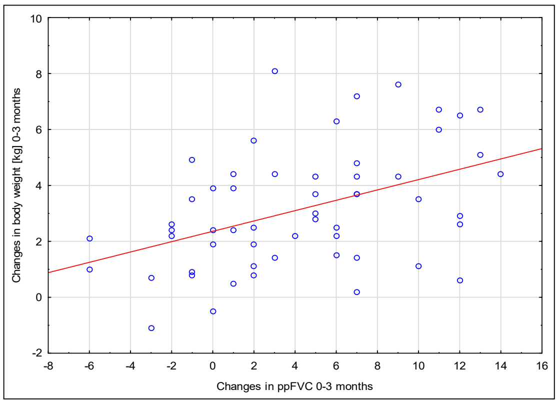

Figure S17. Scatter plot of change in body weight [kg] versus change in ppFVC from baseline to 3 months.

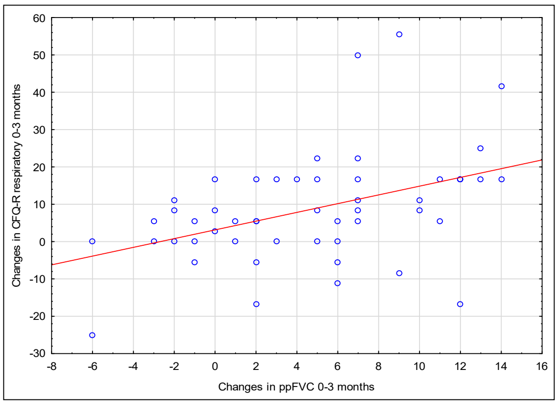

Figure S18. Scatter plot of change CFQ-R Respiratory versus change in ppFVC from baseline to 3 months.

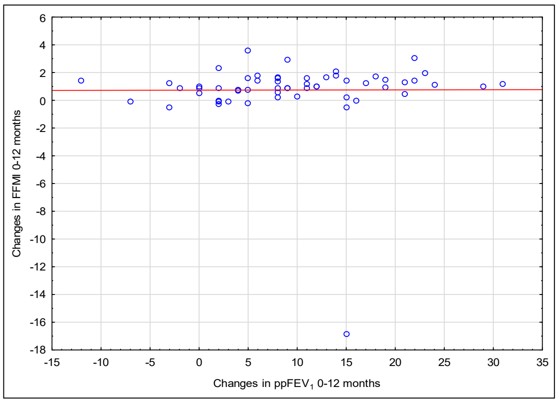

Figure S19. Scatter plot of change inFFMI [kg/m^2] versus change in ppFEV$_1$ from baseline to 12 months.

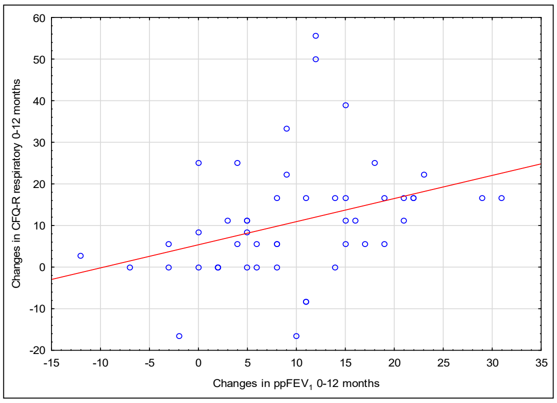

Figure S20. Scatter plot of change in CFQ-R respiratory versus change in ppFEV$_1$ from baseline to 12 months.

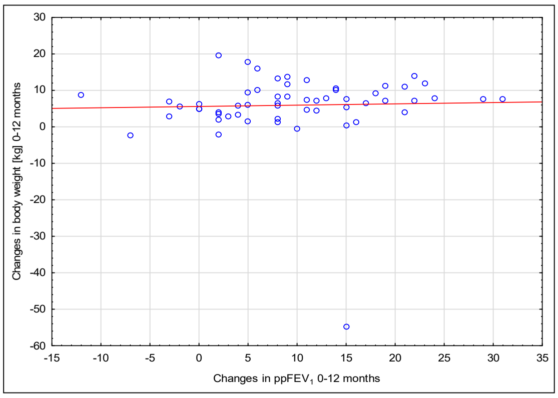

Figure S21. Scatter plot of change in body weight [kg] versus change in ppFEV$_1$ from baseline to 12 months.

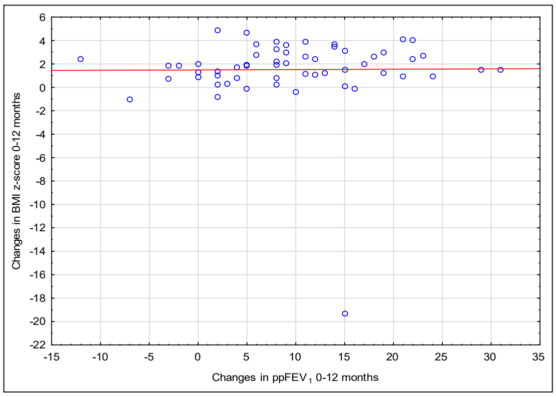

Figure S22. Scatter plot of change in BMI z-score versus change in ppFEV$_1$ from baseline to 12 months.

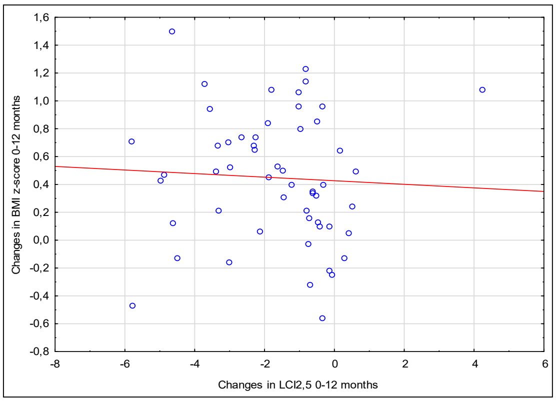

Figure S23. Scatter plot of change in BMI z-score versus change in LCI 2,5 from baseline to 12 months.

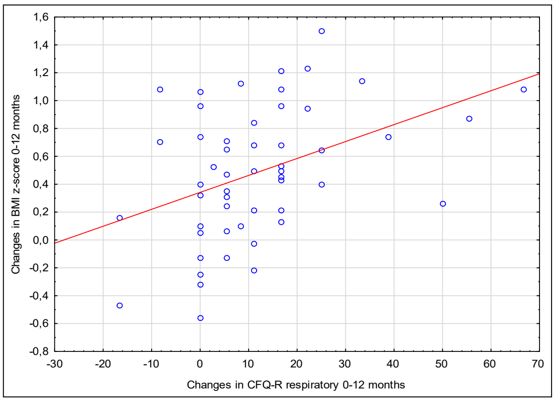

Figure S24. Scatter plot of change in BMI z-score versus change in CFQ-R respiratory from baseline to 12 months.

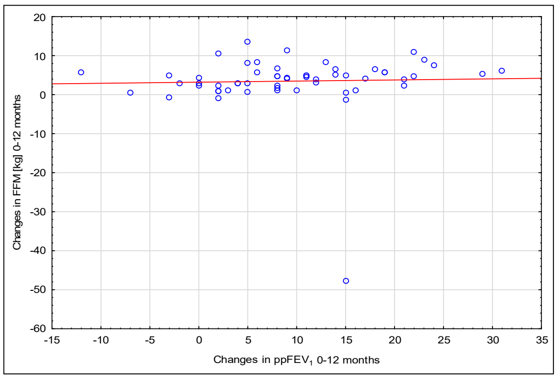

Figure S25. Scatter plot of change in FFM [kg] versus change in $ppFEV_1$ from baseline to 12 months.

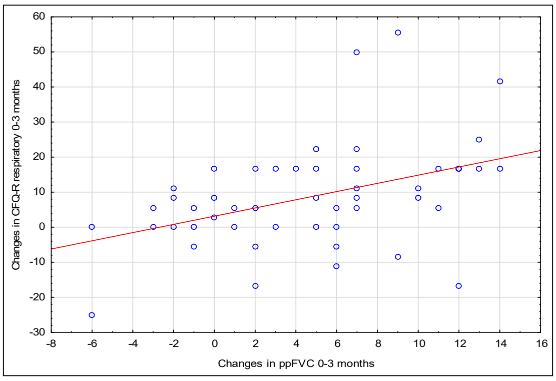

Figure S26. Scatter plot of change in CFQ-R respiratory versus change in ppFVC from baseline to 3 months.

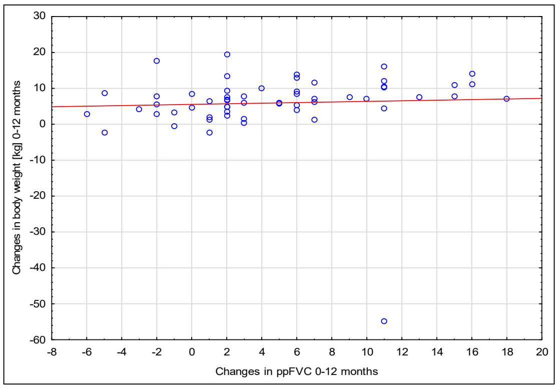

Figure S27. Scatter plot of change in body weight [kg] versus change in ppFVC from baseline to 12 months.

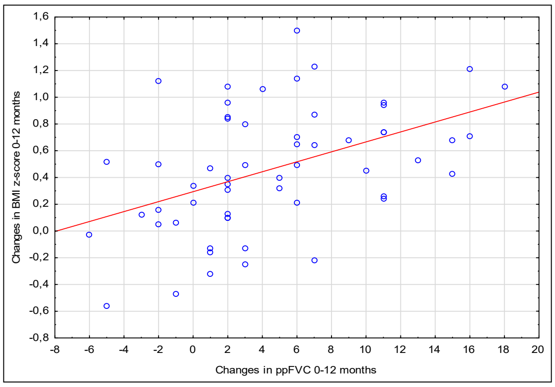

Figure S28. Scatter plot of change in BMI z-score versus change in ppFVC from baseline to 12 months.

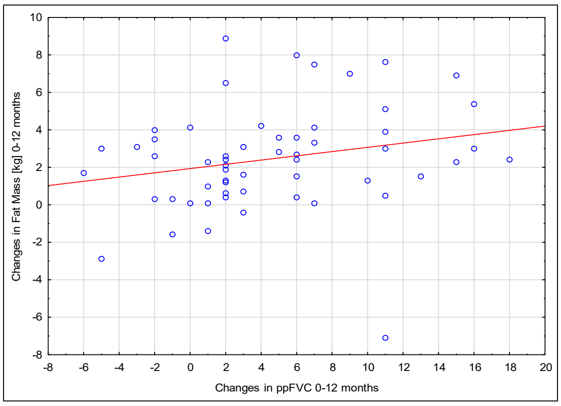

Figure S29. Scatter plot of change in Fat Mass [kg] versus change in ppFVC from baseline to 12 months.

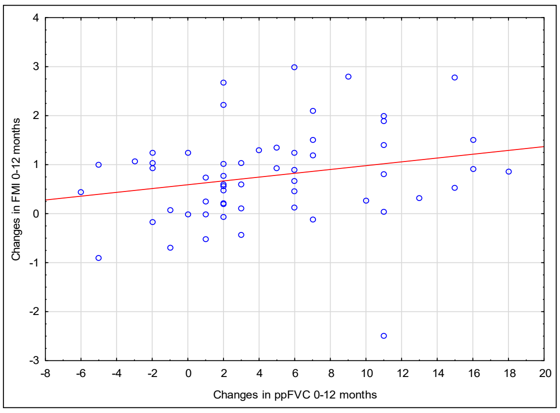

Figure S30. Scatter plot of change in FMI [kg/m^2] versus change in ppFVC from baseline to 12 months.

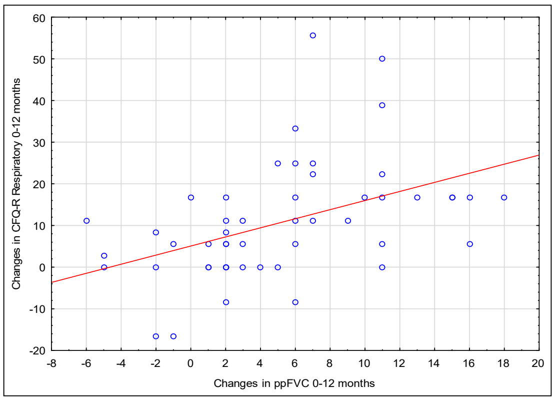

Figure S31. Scatter plot of change in CFQ-R respiratory versus change in ppFVC from baseline to 12 months.

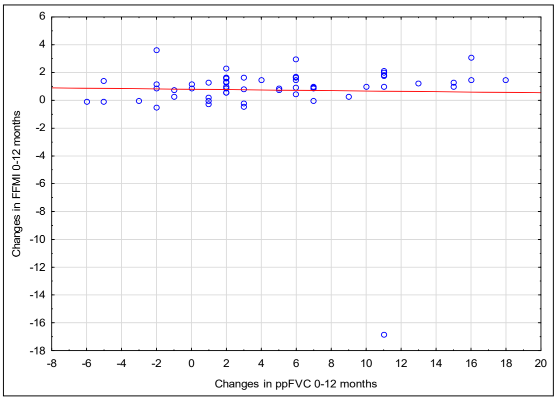

Figure S32. Scatter plot of change in FFMI [kg/m^2] versus change in ppFVC from baseline to 12 months.

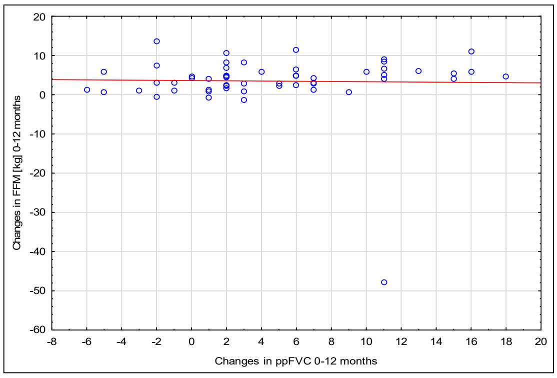

Figure S33. Scatter plot of change in FFM [kg] versus change in ppFVC from baseline to 12 months.
